# Genomic Investigation and Comparative Analysis of European High-Risk Clone of *Acinetobacter baumannii* ST2

**DOI:** 10.3390/microorganisms12122474

**Published:** 2024-12-02

**Authors:** David Hummel, Janos Juhasz, Katalin Kamotsay, Katalin Kristof, Basil Britto Xavier, Sien De Koster, Dora Szabo, Bela Kocsis

**Affiliations:** 1 Institute of Medical Microbiology, Semmelweis University, 1089 Budapest, Hungary; 2 Faculty of Information Technology and Bionics, Pázmány Péter Catholic University, 1083 Budapest, Hungary; 3 Central Microbiology Laboratory, National Institute of Hematology and Infectious Disease, Central Hospital of Southern-Pest, 1097 Budapest, Hungary; 4 Institute of Laboratory Medicine, Semmelweis University, 1083 Budapest, Hungary; 5 Laboratory of Medical Microbiology, University of Antwerp, 2610 Antwerpen, Belgium; 6 Department of Medical Microbiology and Infection Control, University of Groningen, University Medical Center Groningen, 9713 Groningen, The Netherlands; 7 Microbiology Department, Antwerp University Hospital (UZA), 2650 Edegem, Belgium; 8 HUN-REN-SU Human Microbiota Research Group, 1052 Budapest, Hungary; 9 Department of Neurosurgical and Neurointervention, Semmelweis University, 1085 Budapest, Hungary

**Keywords:** antibiotic resistance, *Acinetobacter baumannii*, high-risk clone, nosocomial infection, whole-genome sequencing

## Abstract

Multidrug-resistant *Acinetobacter baumannii* is a major concern in healthcare institutions worldwide. Several reports described the dissemination of *A. baumannii* high-risk clones that are responsible for a high number of difficult-to-treat infections. In our study, 19 multidrug-resistant *A. baumannii* strains from Budapest, Hungary, were investigated based on whole-genome sequencing (WGS). The obtained results were analysed together with data from 433 strains of *A. baumannii* from the Pathogenwatch database. WGS analysis of 19 *A. baumannii* strains detected that 12 belonged to ST2 and seven belonged to ST636. Among ST2 strains, 11 out of 12 carried either *bla*_OXA-23_ or *bla*_OXA-58_ genes; however, all strains of ST636 uniformly carried *bla*_OXA-72_ gene. All strains of ST2 and ST636 carried *bla*_OXA-66_ and *bla*_ADC-25_ genes. Based on core genome multilocus sequence typing (cgMLST), 10 strains of ST2 belonged to cgMLST906, one strain to cgMLST458, and one strain to cgMLST1320; by contrast, all strains of ST636 belonged to cgMLST1178. Certain virulence determinants were present in all strains of both ST2 and ST636, namely, Ata, Bap, BfmRS, T2SS and PNAG. Interestingly, OmpA was present in all strains of ST2, but it was absent in all strains of ST636. Comparative analysis of 19 strains of this study and the collection of 433 isolates from Pathogenwatch database, proved a diverse clonal distribution of high-risk *A. baumannii* clones in Europe. The major clone in Europe is ST2, which is present all over the continent. However, ST636 has been mainly reported in Eastern Europe. Interestingly, cgMLSTs of ST2 correspond to the production of different beta-lactamases, namely, OXA-82 in cgMLST116, OXA-72 in cgMLST506, and cgMLST556, PER-1 in cgMLST456 and cgMLST1041. Our study demonstrates that the ST2 high-risk clone of *A. baumannii* is the most widespread in Europe; however, based on cgMLST analysis, a detailed detection of beta-lactamase production can be determined.

## 1. Introduction

*Acinetobacter baumannii* is a major Gram-negative nosocomial pathogen that poses an increasing threat to inpatients owing to its ability to develop and spread antibiotic resistance and to persist in hospital environments [1]. The rising threat posed by this pathogen results from its genomic plasticity and adaptability [2]. Carbapenem-resistant *A. baumannii* (CRAB) is categorized in the critical group (which signifies the highest importance and urgency) in the WHO Bacterial Priority Pathogens List, 2024. CRAB excels among nosocomial infections, with a high (>30%) mortality and low treatability rate [3]. The most prevalent sites of CRAB infection are the bloodstream, urinary tract, and respiratory tract, which are commonly associated with plastic devices (e.g., indwelling catheters). A particularly serious problem occurs in intensive care units (ICUs), where CRAB is mainly isolated from ventilator-associated pneumonia. CRAB causes complicated infections, which are associated with longer ICU stays, higher hospitalization costs, and higher mortality rates [4,5,6]. Environmental contamination plays an important role in hospitals in the development and persistence of CRAB infections. The transmission of pathogens can occur via air, gloves, medical devices, etc. Moreover, the ability of pathogens to survive in harsh circumstances makes eradication even more difficult. Studies have shown that gastrointestinal and respiratory tract colonization among inpatients is of special importance for environmental contamination [6,7].

*A. baumannii* has an exceptional ability to develop resistance to different classes of antibiotics. Multidrug-resistant *A. baumannii* showed a steadily increasing prevalence in Europe for the years 2017–2021, with one of the highest proportions of multidrug resistance among the pathogens isolated from ICUs [8,9]. According to the surveillance report of the European Centre for Disease Prevention and Control (ECDC), 59.1% of the tested invasive *A. baumannii* isolates were resistant to all three antimicrobial groups under surveillance (fluoroquinolones, aminoglycosides, and carbapenems) by 2022 [10]. In Hungary, the proportion of combined resistance was 44.3%, as reported by the Surveillance Atlas of Infectious Diseases (https://atlas.ecdc.europa.eu (accessed on 12 March 2024)). As stated in the 2022–2023 point prevalence survey of the ECDC, we found that in the European Union (EU), 3.2% of the reported healthcare-associated infections (HAIs) were caused by *A. baumannii*, of which 82.9% were carbapenem-resistant [11].

Carbapenems are broadly used as last-resort antibiotics against nosocomial infections in critically ill patients because of their broad spectrum and outstanding antibacterial efficacy [5]. The most frequent cause of carbapenem resistance is the production of different carbapenem-hydrolyzing enzymes, which are classified into different groups according to their chemical structures. *A. baumannii* most commonly produces Ambler class D carbapenem hydrolyzing enzymes, which have six main groups: intrinsic OXA-51-like and acquired OXA-23-like, OXA-58-like, OXA-24/40-like, OXA-235-like, and OXA-143-like β-lactamases [12]. Additionally, Ambler class B metallo-beta-lactamases, namely NDM and VIM, also occur in carbapenem-resistant *A. baumannii* clinical isolates [6]. According to a broad European study conducted between 2016 and 2018, the predominant carbapenemase gene among CRAB clinical isolates was *bla*_OXA-23_, which was present in 67.7% of the samples. This was followed by *bla*_OXA-72_, which belongs to the OXA-24/40-like group. It is a single amino acid variant of *bla*_OXA-24_ and was present in 30.1% of the samples [13]. The dominance of OXA-23 is not limited to Europe. Another study in 2019 from a global perspective found that 81.8% of the publicly available genomes of *A. baumannii* in the NCBI GenBank database, which contained carbapenem resistance genes, had *bla*_OXA-23_ (1918 genomes out of 2345) [7].

Behind the worldwide spread of *A. baumannii* as a nosocomial pathogen, we found a handful of epidemic clones that caused most cases. According to the Pasteur multilocus sequence typing (MLST) schema, the most prevalent type was ST2 [14], which belongs to the International Clone 2. [5,12]. Studies have found that in hospital wards, certain successful clones can persist for even a decade; however, this does not rule out the dissemination of other clones concurrently, which can lead to polyclonal endemicity [15].

The success of ST2 is probably due to the acquisition of several antibiotic resistance islands (ARIs) [1]. Other mobile genetic elements that often affect the resistance of *A. baumannii* include insertion sequences (IS). There are several types of IS in *A. baumannii*. Yet, the most diffuse is *ISAba1*, which is found upstream of *bla*_OXA-23_, *bla*_OXA-58_, and *bla*_OXA-51_ genes, and it serves as a promoter region to enhance the expression of these beta-lactamase genes, possibly leading to carbapenem resistance [5,12]. Studies regarding the prevalence of *A. baumannii* clones in Eastern Europe also confirmed that ST2 is predominant in this region. In addition, some clones are typical of this geographical region, such as ST1, ST492, and ST636. Regarding OXA-type carbapenemase genes, next to *bla*_OXA-23_, *bla*_OXA-72_ is the most common [16,17,18,19,20,21].

As part of this study, we collected 19 clinical isolates from a central hospital and a clinical hospital in Budapest, Hungary, between 2021 and 2022. We tested the resistance profiles and performed molecular typing and whole-genome sequencing. We did not find a recent study with a European outlook on *A. baumannii* clinical isolates, which takes antibiotic resistance genes and virulence factors into consideration. This would also help us to examine the results obtained from the 19 samples from Budapest in a broader context. Therefore, we collected data on 433 clinical isolates from Europe using the well-curated, and structured Pathogenwatch database (https://pathogen.watch (accessed on 20 February 2022) [22,23]. Focusing on the most prevalent ST2 clone, we examined MLST, core genome MLST (cgMLST), and resistance and virulence profiles of 188 samples, of which 12 were collected by us. Our results help us to understand the spread, resistance genes and virulence determinants of *A. baumannii* ST2 present in Europe.

## 2. Materials and Methods

### 2.1. Strains

We collected 19 multidrug-resistant (MDR) *A. baumannii* clinical isolates from two hospitals in Budapest, Hungary: 10 isolates from the Central Hospital of Southern Pest—National Institute of Hematology and Infectious Diseases (SPC) and 9 isolates from the Semmelweis University Clinical Centre (SU) between 2021 and 2022. Bacterial isolation was performed during routine work in SPC and SU laboratories. Colonies from the samples were cultured at 37 °C for 24 h under aerobic conditions on Columbia blood agar.

The identification of isolates was carried out by Matrix-assisted Laser Desorption/Ionization Time-of-Flight Mass Spectrometry (MALDI-TOF MS) (SU: Bruker Daltonics, Bremen, Germany; SPC: Vitek MS, bioMérieux SA, Marcy l’Étoile, France). The antimicrobial susceptibility testing of *A. baumannii* isolates was performed using disk diffusion for imipenem, meropenem, ciprofloxacin, levofloxacin (only in SU), sulfamethoxasole/trimethoprim, amikacin, tobramycin, and gentamicin; except for colistin, which was performed by broth microdilution method. The obtained results were interpreted according to the EUCAST guidelines protocol v14.0 (www.eucast.org (accessed on 12 January 2024)) [22] (Table S1: Metadata of the 19 isolates of this study).

### 2.2. Whole-Genome Sequencing

Genomic DNA of 19 *A. baumannii* strains was extracted by MasterPure DNA Purification Kit (Epicentre Technologies Inc., Madison, WI, USA). DNA quality and concentration were measured using Qubit fluorometer (Thermo Fisher Scientific, Waltham, MA, USA) and NanoDrop (Thermo Fisher Scientific, Waltham, MA, USA). Library preparation was performed with Nextera XT sample preparation kit (Illumina, San Diego, CA, USA).

The whole-genome sequencing (WGS) was performed with 2 × 250 bp paired-end sequencing using the Illumina MiSeq platform (Illumina, San Diego, CA, USA) at the University of Antwerp. Sequences were submitted to the NCBI BioProject database (BioProject ID: PRJNA1173775).

### 2.3. Genome Sequence Analysis

The genomic analysis was carried out by BacPipe (v1.2.6) [23] software, which includes other programs such as SPAdes [24] (v3.13.0) for short-read assembly and Prokka (v1.12) [25] for genome annotation. BacPipe comprises searches in different databases, such as PubMLST [26] for MLST typing and ResFinder [27] for antibiotic resistance gene (ARG) prediction. For the MLST typing, we used the “Pasteur” 7 loci MLST scheme (MLST v2.0) [28], and because of the higher discriminatory power (2133 loci), we also adopted the core genome MLST (cgMLST) typing (cgMLSTFinder 1.2) [29,30,31,32].

Compared to results provided by BacPipe, we aimed to conduct a more comprehensive study regarding virulence factors; therefore, we screened the genomes using the ABRicate program (https://github.com/tseemann/abricate (accessed on 20 February 2022)) for virulence factors from the VFDB database [33] and from other sources [34,35] (Table S2: List of analysed virulence factors with their category and related genes). Regarding the categorization of virulence factors, we applied the general classification scheme for bacterial virulence factors introduced into VFDB in 2022 [36]. We regarded a gene as present when at least 90% of the gene was covered.

To understand and visualize the genetic relatedness between the isolates, we used the goeBURST algorithm provided by the PHYLOViZ Online tool (https://online.phyloviz.net/ (accessed on 20 February 2022)) [37,38] to produce minimum spanning tree graphs. We also used the Rawgraphs (https://www.rawgraphs.io/ (accessed on 20 February 2022)) open-source online tool to create circle-packing figures.

### 2.4. Analysis of A. baumannii Strains from Pathogenwatch

We selected *A. baumannii* isolates detected in Europe from the Pathogenwatch database. We filtered out environmental and non-human samples and those lacking information on the source of isolation. Therefore, we identified a total of 433 clinical isolates (Table S3: Metadata of the 433 isolates from Pathogenwatch). After the analysis, we observed the widespread dominance of ST2 and focused our research on this. After eliminating samples from the same outbreak, 176 isolates remained (Table S4: Metadata of the 176 ST2 isolates from Pathogenwatch).

### 2.5. Statistical Analysis

In this study online programs were used to analyse genomic data. The VFDB database and ABRicate software v0.9.8 were used to identify virulence factors genes. BacPipe software identified the virulence factor in the VirDB database. Virulence factors were identified with ABRicate by obtaining the sequence from the NCBI RefSeq database.

## 3. Results

### 3.1. WGS Analysis, Antibiotic Resistance Profile of the 19 A. baumannii

**Figure 1:**
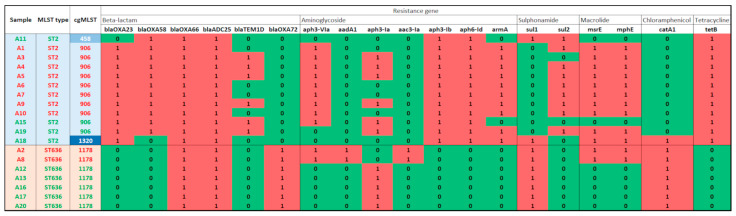
Detected antibiotic resistance genes in 19 *A. baumannii* strains of ST2 and ST636.

**Figure 2:**
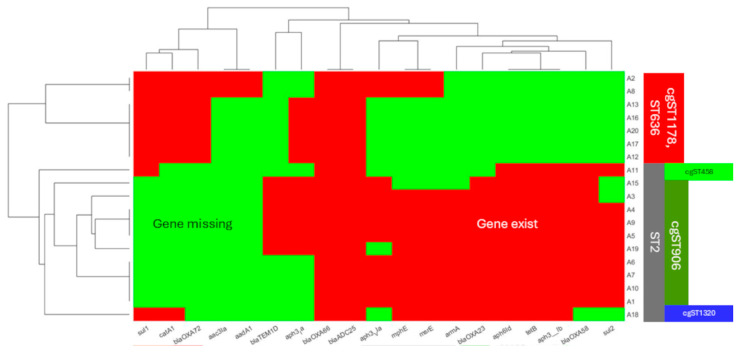
Distribution of antibiotic resistance genes (ARGs) in 19 *A. baumannii* strains of ST2 and ST636.

All 19 *A. baumannii* strains were investigated by WGS and the obtained results are demonstrated in Figure 1. Out of the 19 strains, 12 belonged to ST2, and seven belonged to ST636. Interestingly, diverse antibiotic resistance patterns were detected in *A. baumannii* strains of this study. Ten ST2 strains contain both *bla*_OXA-23_ and *bla*_OXA-58_, while 11 ST2 strains contain either *bla*_OXA-23_ or *bla*_OXA-58_, by contrast, all strains of ST636 uniformly carried *bla*_OXA-72_. OXA-66 and ADC-25 were present in all strains of ST2 and ST636. Aminoglycoside resistance genes distributed among strains of ST2 and ST636 as follows: *aph(3′)-Ia* and *aph(3′)-VIa* were present in strains of ST2 and ST636; *aph(3′)-Ib*, *aph(6′)-Id*, *armA* were present in strains of ST2, while *aac(3′)-Ia* and *aadA1* were detected in strains of ST636. The gene *tet(B)*, which is responsible for tetracycline resistance, was found in all ST2 samples but not in ST636 isolates. A distribution of *sul1* and *sul2* was determined, and interestingly *sul1* was predominantly present in strains of ST636, while, on the other hand, *sul2* was present mainly in strains of ST2. All the detected ARGs in strains of ST2 and ST636 are shown in Figure 1 and Figure 2, along with the cgMLST classification.

### 3.2. Virulence Profile of the Isolates

**Figure 3:**
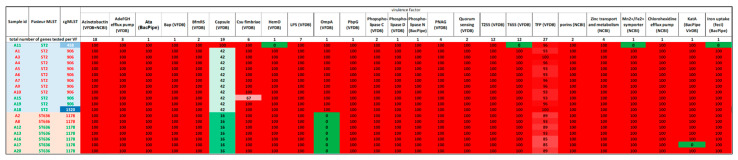
The proportion of virulence genes present in the 19 *A. baumannii* genomes of this study was compared with the number of all tested genes per virulence factor (VF). Abbreviations: Ata: Acinetobacter trimeric autotransporter, Bap: Biofilm-associated protein, OmpA: Outer membrane protein A, PbpG: Penicillin-binding protein, T2SS: Type II secretion system, T6SS: Type VI secretion system, TFP: Type IV pili, KatA: catalase. In parentheses: ‘VFDB’: The VFDB database and ABRicate software were utilized to identify these genes; ‘BacPipe’: The gene was identified in the gbk file of BacPipe output; ‘BacPipe VirDB’: BacPipe software identified the virulence factor in the VirDB database; ‘NCBI RefSeq’: These virulence factors were identified with ABRicate by obtaining the sequence from the NCBI RefSeq database.

**Figure 4:**
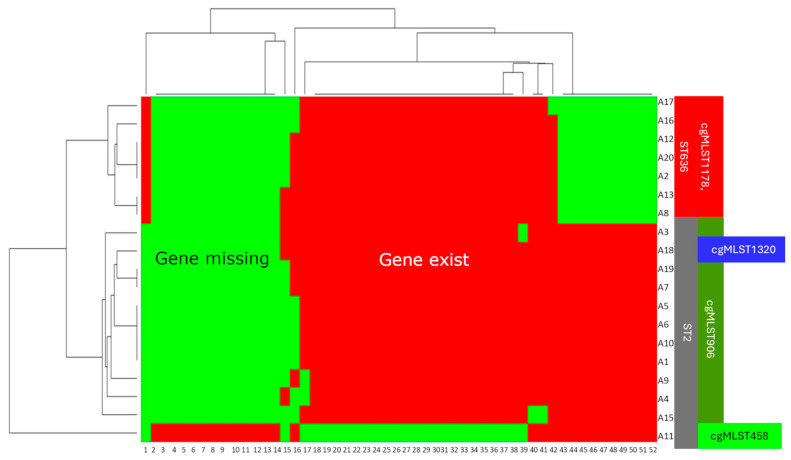
Distribution of a subset of differentiating virulence genes among the isolates. The numbers along the horizontal axis denote virulence genes as follows: 1: *bauC* (NCBI RefSeq), 2: capsule gene, 3: *plcN*, 4: *pilA* (NCBI RefSeq), 5: capsule gene, 6: capsule gene, 7: capsule gene, 8: *pseB*, 9: *pseC*, 10: *pseF*, 11: *pseG*, 12: *pseH*, 13: *pseI*, 14: *tviB*, 15: *pilS*, 16: *pilI*, 17: capsule gene, 18: capsule gene, 19: *vgrG/tssI*, 20: *mntH*, 21: *fecI*, 22: *tssM*, 23: capsule gene, 24: *tssK*, 25: capsule gene, 26: *tssG*, 27: capsule gene, 28: capsule gene, 29: capsule gene, 30: *hemO*, 31: *clpV*/*tssH*, 32: *hcp/tssD*, 33: *tagX*, 34: *tssA*, 35: *tssB*, 36: *tssC*, 37: *tssE*, 38: *tssF*, 39: capsule gene, 40: *csuD*, 41: *csuE*, 42: *katA*, 43: *pilA* (VFDB), 44: capsule gene, 45: *ompA*, 46: *pgi*, 47: capsule gene, 48: capsule gene, 49: capsule gene, 50: capsule gene, 51: *abaI* (NCBI RefSeq), and 52: *bauA*.

We conducted extensive research on virulence factors by identifying genes related to virulence factors (Figure 3). The values represent the percentage of identified genes as a proportion of all genes tested for each group of virulence factors. Regarding the virulence factors, the samples were similar to each other. Relevant differences were found at OmpA, which was present in all ST2 isolates but was absent in all ST636 isolates according to our research. OmpA is a significant and widely studied virulence factor in *A. baumannii* that plays a role in adherence and antibiotic resistance [39]. In addition, a peculiarity of cgMLST458, which belongs to ST2, was observed. It lacks iron-uptake systems, for example, HemO, which is characteristic of hypervirulent *A. baumannii* strains [40], and the type VI secretion system is absent. The distinctiveness of cgMLST458 is shown in Figure 4, along with the different capsular protein characteristics of ST636.

### 3.3. cgMLST Allelic Profiles and Phylogenetic Relationships

**Figure 5:**
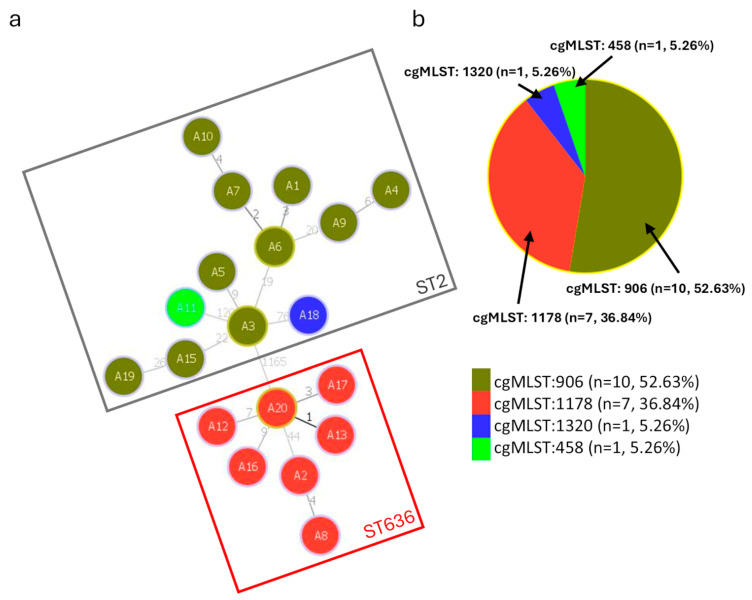
(**a**) Minimum spanning tree based on core genome multilocus sequence typing (cgMLST) allelic profiles of 19 *A. baumannii* strains of this study. The numbers on the connecting lines indicate the allelic differences. (**b**) cgMLST distribution of the samples. Various cgMLSTs are represented by different colours.

Based on the cgMLST allelic profiles of 2133 loci, a minimum spanning tree of the 19 isolates of this study was generated (Figure 5). Remarkably, the 19 isolates can be clustered into two major phylogenetic groups, with large genetic distances between the group of ST2 and the group of ST636 strains. Within the ST636 group, we observed relative genetic homogeneity, such that all seven isolates belonged to the same cgMLST. In contrast, in the ST2 strain group, we found three different cgMLST subgroups, two of which were singletons (cgMLST458 and cgMLST1320), whereas cgMLST906 included 10 isolates. Considering that the 19 strains of the study were collected from two different hospitals, isolates from both hospitals were present in both ST2 and ST636 groups.

**Figure 6:**
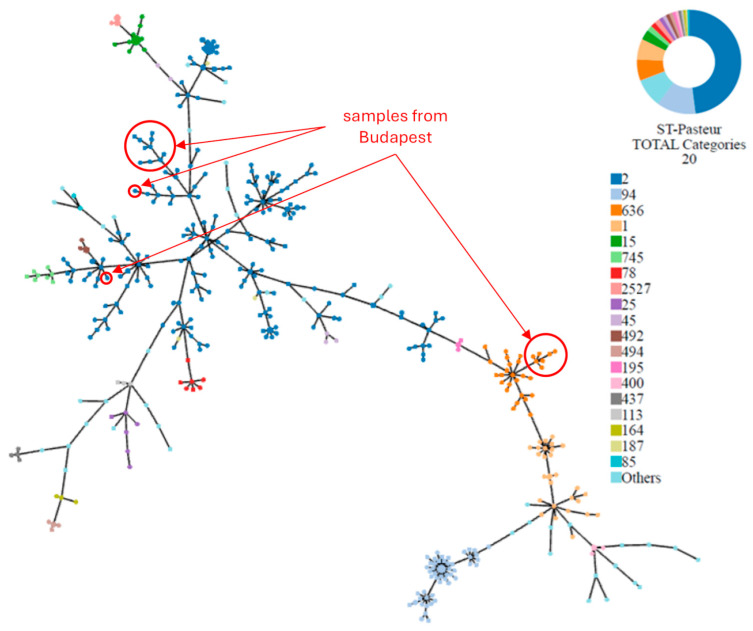
Phylogenetic tree based on cgMLST allelic loci profiles of 452 (433 + 19) isolates from Europe. Diverse sequence types (STs) are distinguished by their different colours. Samples from Budapest refer to the 19 *A. baumannii* isolated in this study.

To gain a broader perspective on our samples, we constructed a graph based on the cgMLST allelic profiles of our 19 samples and the 433 *A. baumannii* clinical isolates from Pathogenwatch, where we marked the different “Pasteur” STs with different colours (Figure 6). Among these 452 (433 + 19) European isolates, ST2 (47.8%) was the most prevalent, other significant STs were ST94 (12.2%), ST636 (6.6%), ST1 (6.4%), and ST15 (3.1%).

**Figure 7:**
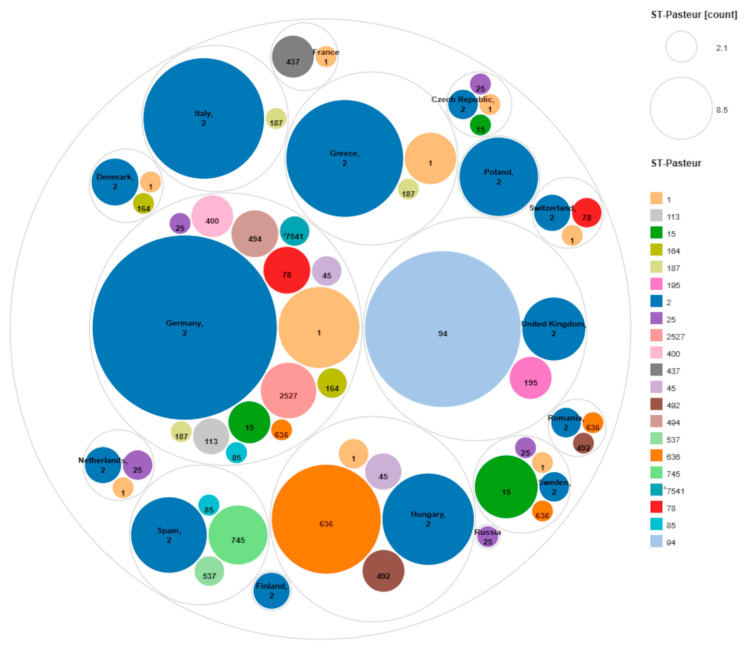
Circle packing figure of 414 non-singleton isolates out of 452 (433 + 19) from Europe. The outer circles represent the country of origin, whereas the inner circles represent the STs. The size of the circles is proportional to the number of represented isolates in Pathogenwatch, the asterisk indicates a novel ST that was not classified in the database.

Figure 7 shows 414 non-singleton isolates out of the total 452 (433 + 19) analysed isolates grouped according to their country of origin and their corresponding ST. As the size of the circles corresponds to the number of isolates, the high prevalence of ST2 in Europe was obvious. In addition, from the most relevant strains ST94 was from the United Kingdom, ST636 mainly from Hungary, ST1 can be found overwhelmingly in Germany and Greece, while ST15 is found primarily in Sweden.

### 3.4. Study of ST2 Isolates

Owing to its dominance and ubiquity, we focused our analysis on ST2. Out of the 433 isolates from Pathogenwatch, 176 belonged to ST2, and we also analysed our 12 isolates of ST2.

**Figure 8:**
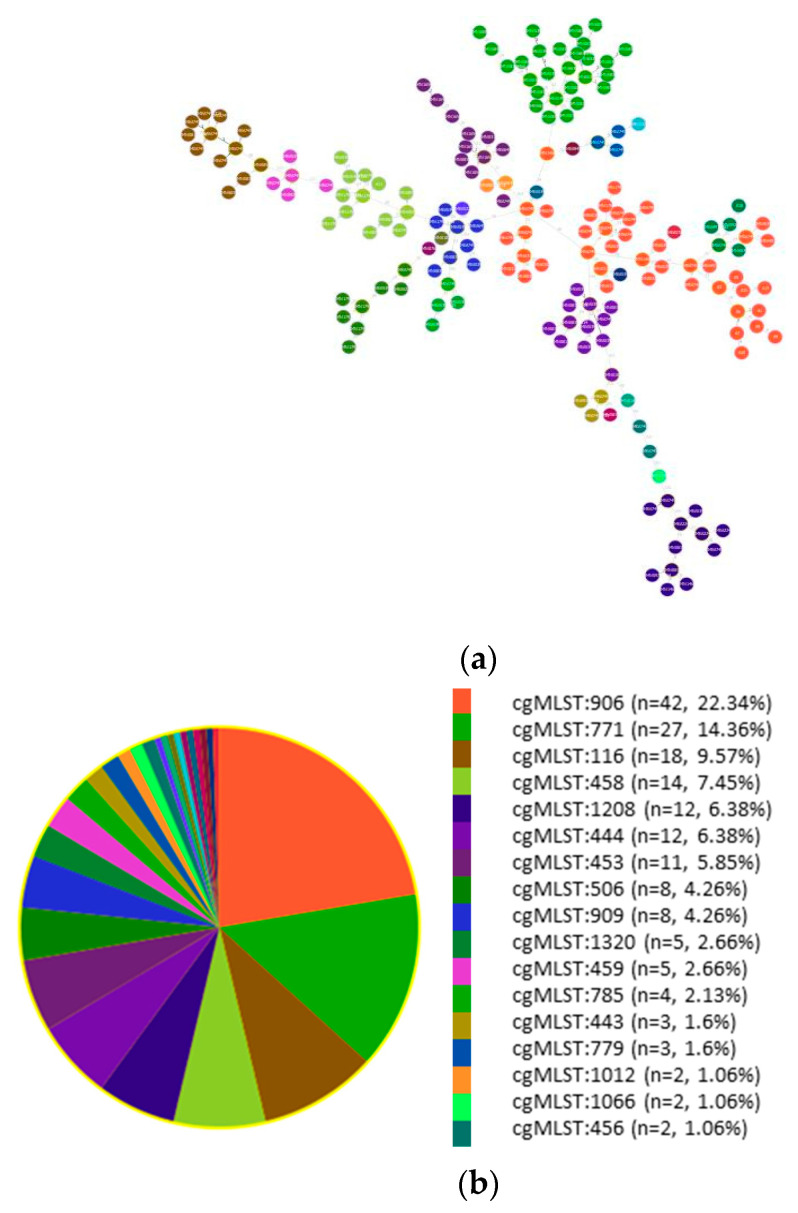
(**a**) Phylogenetic relationship between European 188 (176 + 12) ST2 isolates based on cgMLST allelic profiles. The legend shows the non-singleton cgMLSTs. (**b**) cgMLST distribution of the 188 samples. Various cgMLSTs are represented by different colours.

Figure 8 shows the cgMLSTs of the isolates belonging to ST2 and their genetic relatedness in a phylogenetic tree. The most common cgMLST in Europe was cgMLST906 among the Pathogenwatch isolates and in our samples. The 12 ST2 samples in our study belonged to cgMLST906 (10 strains), cgMLST458 (one strain) and cgMLST1320 (one strain). Interestingly, cgMLST458 and cgMLST1320 were present in our samples, but these were found less frequently in the European isolates (Figure 8).

**Figure 9:**
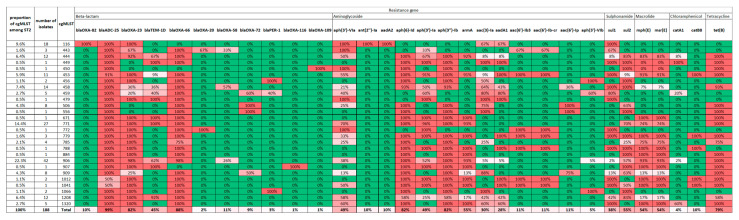
Distribution of ARGs among the cgMLSTs of ST2. Percentages represent the proportion of isolates within a certain cgMLST containing that gene. The last row indicates the overall proportion of this gene among all 188 ST2 isolates, while the first column shows the proportion of certain cgMLSTs. Red colour indicates 100%, green colour indicates 0% prevalence among analysed cgMLST isolates.

It proved interesting to examine the distribution of ARGs among ST2 isolates using BacPipe. Due to the large number of isolates, we present our findings according to cgMLSTs. The results are shown in Figure 9. The most widespread ARGs were *bla*_ADC-25_ and *bla*_OXA-66_; however, *bla*_OXA-23_, *aph(6)-Id*, *aph(3″)-Ib*, and *tet(B)* were also common. In the largest cgMLST906 group all these genes were typically present, as well as *armA* and the macrolide-related *mph(E)* and *msr(E)*, respectively. Among the 10 cgMLST906 isolates of this study, nine had this previously described characteristic ARG profile, except for sample A15, which lacked the *armA*, *msr(E)*, and *mph(E)* genes. With reference to cgMLST458 and cgMLST1320, which were present as singletons among the 19 samples of this study, we can mark that the ARGs present in these samples were in accordance with the ARG profile of their cgMLST group.

**Figure 10:**
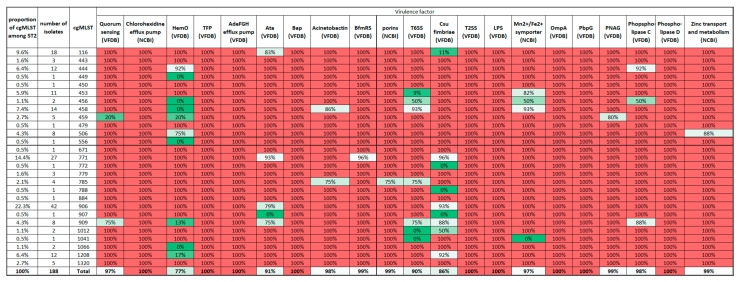
Distribution of virulence factors among cgMLSTs of ST2. Percentages represent the proportion of isolates within a certain cgMLST containing at least 90% of the virulence genes belonging to that virulence factor. In this case, we assume that a given virulence factor is present and functioning. The last row indicates the overall proportion of samples with the corresponding virulence factor. Abbreviations: Ata: Acinetobacter trimeric autotransporter, Bap: Biofilm-associated protein, OmpA: Outer membrane protein A, PbpG: Penicillin-binding protein, T2SS: Type II secretion system, T6SS: Type VI secretion system, TFP: Type IV pili, KatA: catalase. In parentheses: ‘VFDB’: The VFDB database and ABRicate software were utilized to identify these genes; ‘NCBI RefSeq’: These virulence factors were identified with ABRicate by obtaining the sequence from the NCBI RefSeq database. Red colour indicates 100%; green colour indicates 0% prevalence among analysed cgMLST isolates.

Similarly, we examined the presence of virulence factors among the cgMLSTs, the results are shown in Figure 10. As it can be seen, the great majority of virulence factors are present in all samples. (In this figure, the data regarding capsule genes are not included.) Interestingly, the presence of HemO varied among the ST2 samples and Csu fibriae, Ata, and T6SS to a certain degree.

## 4. Discussion

*A. baumannii* remains a major challenge in hospitals due to the asymptomatic carriage of patients and staff as well as due to the long-lasting survival of this pathogen in hospital environment. It has been recognized in several countries that colonization and transmission of MDR *A. baumannii* can take place easily; therefore, it can cause large outbreaks that are associated with high mortality rates [17,19]. It is of utmost importance to improve the investigation of MDR *A. baumannii* strains by utilizing WGS to detect the circulating clones and to analyse their ever-growing resistance profiles. Based on WGS data, the online databases can be regularly updated, and the new available data can be enhanced to combat outbreaks caused by MDR *A. baumannii* [3,11]. In our study, we have demonstrated that online databases, such as Pathogenwatch, are very useful tools in analyzing MDR *A. baumannii* strains in Europe. The regularly updated online databases can help infection control teams and stakeholders in hospitals to improve infection control measures, antibiotic stewardship and antibiotic policy in order to effectively prepare hospital staff to cope with future outbreaks. Multidrug-resistant *A. baumannii* strains have disseminated worldwide and these strains represent a major challenge and concern in hospitals, especially in ICUs [6].

Among MDR *A. baumannii* strains, high-risk clones are capable of developing multidrug resistance, persisting in hospital environments, and retaining their fitness. Therefore, these clones are responsible for a high number of difficult-to-treat infections [41]. In our study, ST2 and ST636 clones were analysed based on WGS data, and a comparative analysis was performed based on available genomic data of ST2 clones in the Pathogenwatch database.

ST2 is a widely disseminated international high-risk clone. It has been previously reported [7] to be the most dominant ST belonging to international clone 2 (IC2) [42]; therefore, the study of this type has particular relevance.

ST636 has been mainly reported in Eastern European countries (Bulgaria [43], Croatia [44], Serbia [45], Albania [21], Romania [19]) and in Colombia [46]. Previously, both ST2 and ST636 were reported in Hungary [16].

In this study, 19 *A. baumannii* strains were evaluated based on ST, cgMLST, ARG and virulence gene profiles. Differential ARG profiles were found between the two sequence types, ST2 and ST636 samples. The latter carried *bla*_OXA-72_, whereas ST2 isolates harbored *bla*_OXA-23_, except for one strain. These two STs clustered into two phylogenetic groups based on the cgMLST allelic profiles. This difference was also mirrored in the virulence genes, although to a lesser extent; for example, OmpA was not detected in ST636.

The presence of *sul1* and *sul2* genes provides resistance to sulfamethoxasole/trimethoprim. These genes can be mediated via transposons and plasmids [47]. Among our 19 strains of *A. bamumannii* the *sul1* was dominantly present in strains of ST636, but the *sul2* was mainly present in strains of ST2. However, when we analyse 188 strains of ST2, no striking differences can be seen in correlation with *sul* determinant and cgMLST of ST2 (Figure 9). Our findings are in accordance with other studies, since *sul1* and *sul2* determinants were observed to be ubiquitous and mutually exclusive in certain STs, while other STs exhibited a mixture of both [1].

To obtain a broader outlook, 433 European *A. baumannii* isolates were collected from the Pathogenwatch database and subsequently analysed. A cgMLST study was conducted on the samples to determine phylogenetic relationships together with the 19 samples of this study. The analysis based on the countries revealed that certain STs are characteristic of certain countries or regions, such as ST636 to Hungary, whereas the most widespread ST2 is almost omnipresent. Our findings confirmed earlier results that different clonal lineages can persist simultaneously, even in the same hospital [15].

Focusing on the global high-risk clone ST2, we analysed 176 genomes from Pathogenwatch and 12 samples of this study. The cgMLST classification revealed the high prevalence of cgMLST906 in both our and Pathogenwatch samples. The profiles of the isolates were determined after screening for ARGs and virulence genes. The majority (82%) of ST2 isolates carried the widely reported *bla*_OXA-23_ gene, which plays an important role in conferring carbapenem resistance [41]. Our results confirmed that MDR is typical for ST2 clinical isolates [14].

The virulence patterns of the ST2 isolates were more homogenous than those of the ARGs, but the presence of some virulence factors distinguished the samples, such as the presence of HemO or T6SS. It is not surprising that the most successful cgMLST906 strain appeared to have these virulence factors.

Including a large number of European isolates (452 in total) provides a unique value to our study, as long as it offers the possibility to gain a broader look at the European landscape of *A. baumannii* clinical isolates. We put emphasis on the analysis of an extensive set of virulence factors, which contributes a particular significance to our research. The results give the possibility to determine which STs are typical in each European country and to investigate the connections between ARGs, virulence factors, and cgMLSTs among ST2 isolates.

For further studies, we would like to study mobile genetic elements [48] along with additional efflux pump genes [49] in the genome of the isolates, as they are important in antibiotic resistance. Pathogenwatch is a reliable database, but to gain an even more expanded view of the *A. baumannii* landscape, including other databases in the research seems beneficial for the future.

## 5. Conclusions

We conclude that *A. baumannii* represents a great challenge in hospitals due to the ability to develop multidrug resistance and to survive in the environment. In our study, a comparative analysis demonstrated the pattern of clonal distribution of European multidrug-resistant *A. baumannii* that correlated with virulence factors and antibiotic resistance determinants. The predominant *A. baumannii* multidrug-resistant high-risk clone is ST2 in Europe. However, certain diversity can be detected in ST2 according to cgMLSTs. Notably, certain beta-lactamase production corresponds to cgMLSTs of ST2, namely, OXA-82 in cgMLST116; OXA-72 in cgMLST506 and cgMLST556; PER-1 in cgMLST456 and cgMLST1041. Analysis based on cgMLST provides a more accurate detection in correlation with antibiotic resistance genes and virulence factor genes. In our study, we observed that the most prevalent cgMLST906 had nearly all studied virulence factor genes; by contrast, among the studied ST2 isolates regarding virulence factors, the largest heterogeneity was observed for HemO, T6SS, and Csu fimbriae. WGS analysis of MDR *A. baumannii* strains is a very important tool to detect the circulating clones and to analyse their ever-growing antibiotic resistance patterns.

## Data Availability

All genome sequence data are submitted to NCBI database. Bioproject number: PRJNA1173775.

## Supplementary Materials

The following supporting information can be downloaded at: https://www.mdpi.com/article/10.3390/microorganisms12122474/s1, Table S1: Metadata of the 19 isolates of this study; Table S2: List of analysed virulence factors with their category and related genes; Table S3: Metadata of the 433 isolates from Pathogenwatch; Table S4: Metadata of the 176 ST2 isolates from Pathogenwatch.
