# Supplementary material for: Genomic Investigation and Comparative Analysis of European High-Risk Clone of Acinetobacter baumannii ST2
Source: Microorganisms. 2024 Dec 2;12(12):2474. doi: 10.3390/microorganisms12122474 (PMC11728346; doi:10.3390/microorganisms12122474)
Supplement: Supplementary file 1 [file microorganisms-12-02474-s001.zip › microorganisms-3318017-supplementary.pdf]

Table S1: Metadata of the 19 isolates of this study

BioSample accession id	id2	isolate date	type	Imipenem	Meropenem	Ciprofloxacin	Levofloxacin	Sulfamethoxasole/ trimethoprim	Amikacin	Tobramycin	Gentamicin	Colistin	Comment
SAMN44319550	A1	2021	BC	R	R	R	-	R	R	R	R	S	
SAMN44319551	A2	2021	BC	R	R	R	-	R	R	S	R	S	
SAMN44319552	A3	2021	BC	R	R	R	-	R	R	R	R	S	
SAMN44319553	A4	2021	BC	R	R	R	-	R	R	R	R	S	
SAMN44319554	A5	2021	BC	R	R	R	-	R	R	R	R	S	
SAMN44319555	A6	2021	BC	R	R	R	-	R	R	R	R	S	
SAMN44319556	A7	2021	BC	R	R	R	-	R	R	R	R	S	
SAMN44319557	A8	2021	BC	R	R	R	-	R	R	S	R	S	
SAMN44319558	A9	2021	BC	R	R	R	-	R	R	R	R	S	
SAMN44319559	A10	2021	BC	R	R	R	-	R	R	R	R	S	
SAMN44319560	A11	2022	BAL	R	R	R	R	R	S	S	R	S	
SAMN44319561	A12	2022	BAL	R	R	R	R	R	S	S	R	S	
SAMN44319562	A13	2022	BAL	R	R	R	R	R	S	S	R	S	
SAMN44319563	A15	2022	nasal discharge	R	R	R	R	R	R	R	R	S	
SAMN44319564	A16	2022	BAL	R	R	R	R	R	S	S	S	R	# same patient 14 days later
SAMN44319565	A17	2022	BAL	R	R	R	R	R	S	S	S	S	
SAMN44319566	A18	2022	tracheal secretion	R	R	R	R	R	R	R	R	S	
SAMN44319567	A19	2022	urine	R	R	R	R	R	R	R	R	S	
SAMN44319568	A20	2022	tracheal secretion	R	R	R	R	R	S	S	S	S	# same patient 1. day

Table S2: List of analyzed virulence factors with their category and related genes (for the complete set of gene id on VFDB see Pathogenomics of Acinetobacter (http://www.mgc.ac.cn/cgi-bin/VFs/compvfs.cgi?Genus=Acinetobacter)

Virulence factors	Related genes	Tool and source for identificatiion
Adherence		
**Ata**	ata	BacPipe gbk fiile
**TFP**	pilM	ABRicate/VFDB
	pilN	ABRicate/VFDB
	pilO	ABRicate/VFDB
	pilP	ABRicate/VFDB
	pilQ	ABRicate/VFDB
	pilT	ABRicate/VFDB
	pilU	ABRicate/VFDB
	pilF	ABRicate/VFDB
	pilA	ABRicate/VFDB
	pilA	ABRicate/NCBI RefSeq
	pilB	ABRicate/VFDB
	pilC	ABRicate/VFDB
	gspO/pilD	ABRicate/VFDB
	tsaP	ABRicate/VFDB
	fimU	ABRicate/VFDB
	pilV	ABRicate/VFDB
	pilW	ABRicate/VFDB
	pilX	ABRicate/VFDB
	pilY1	ABRicate/VFDB
	-	ABRicate/VFDB
	pilE	ABRicate/VFDB
	fimT	ABRicate/VFDB
	pilG	ABRicate/VFDB
	pilH	ABRicate/VFDB
	pilI	ABRicate/VFDB
	pilJ	ABRicate/VFDB

	pilS	ABRicate/VFDB
	pilR	ABRicate/VFDB
Effector delivery system		
**T2SS**	gspC	ABRicate/VFDB
	gspD	ABRicate/VFDB
	gspE1	ABRicate/VFDB
	gspE2	ABRicate/VFDB
	gspF	ABRicate/VFDB
	gspG	ABRicate/VFDB
	gspH	ABRicate/VFDB
	gspI	ABRicate/VFDB
	gspK	ABRicate/VFDB
	gspL	ABRicate/VFDB
	gspM	ABRicate/VFDB
	gspO/pilD	ABRicate/VFDB
**T6SS**	vgrG/tssI	ABRicate/VFDB
	tssM	ABRicate/VFDB
	tagX	ABRicate/VFDB
	tssE	ABRicate/VFDB
	tssF	ABRicate/VFDB
	tssG	ABRicate/VFDB
	tssK	ABRicate/VFDB
	hcp/tssD	ABRicate/VFDB
	tssB	ABRicate/VFDB
	tssC	ABRicate/VFDB
	tssA	ABRicate/VFDB
	clpV/tssH	ABRicate/VFDB
Exotoxin		
**Phospholipase C**	plc1	ABRicate/VFDB
	plc2	ABRicate/VFDB
**Phospholipase D**	plcD	ABRicate/VFDB
**Phospholipase N**	plcN	BacPipe gbk file
Immune modulation		
**Capsule**	-	ABRicate/VFDB
**LPS**	lpxM	ABRicate/VFDB

	lpxL	ABRicate/VFDB
	lpsB	ABRicate/VFDB
	lpxA	ABRicate/VFDB
	lpxB	ABRicate/VFDB
	lpxC	ABRicate/VFDB
	lpxD	ABRicate/VFDB
**Outer Membrane Proteins (porins)**	ompA	ABRicate/VFDB
	carO	ABRicate/NCBI RefSeq
	omp33-36	ABRicate/NCBI RefSeq
**Chlorohexidine efflux pump**	aceI	ABRicate/NCBI RefSeq
**PbpG**	pbpG	ABRicate/VFDB
Biofilm		
**AdeFGH efflux pump**	adeF	ABRicate/VFDB
	adeG	ABRicate/VFDB
	adeH	ABRicate/VFDB
**Bap**	bap	ABRicate/VFDB
**Csu fimbriae**	csuA/B	ABRicate/VFDB
	csuA	ABRicate/VFDB
	csuB	ABRicate/VFDB
	csuC	ABRicate/VFDB
	csuD	ABRicate/VFDB
	csuE	ABRicate/VFDB
**PNAG**	pgaA	ABRicate/VFDB
	pgaB	ABRicate/VFDB
	pgaC	ABRicate/VFDB
	pgaD	ABRicate/VFDB
**Quorom sensing**	abaI	ABRicate/VFDB
	abaI	ABRicate/NCBI RefSeq
	abaR	ABRicate/VFDB
Nutritional/Metabolic factor		
**Acinetobactin**	basJ	ABRicate/VFDB
	basI	ABRicate/VFDB
	basH	ABRicate/VFDB
	barB	ABRicate/VFDB
	barA	ABRicate/VFDB

	basG	ABRicate/VFDB
	basF	ABRicate/VFDB
	entE	ABRicate/VFDB
	basD	ABRicate/VFDB
	basC	ABRicate/VFDB
	bauA	ABRicate/VFDB/NCBI RefSeq
	bauB	ABRicate/VFDB
	bauE	ABRicate/VFDB
	bauC	ABRicate/VFDB
	bauC	ABRicate/NCBI RefSeq
	bauD	ABRicate/VFDB
	basB	ABRicate/VFDB
	basA	ABRicate/VFDB
	bauF	ABRicate/VFDB
**HemO**	hemO	ABRicate/VFDB
**Mn2+/Fe2+ symporter**	mntH	ABRicate/NCBI RefSeq
**Zinc transporter**	znuB	ABRicate/NCBI RefSeq
	znuC	ABRicate/NCBI RefSeq
	znuD	ABRicate/NCBI RefSeq
**Zinc metabolism**	zigA	ABRicate/NCBI RefSeq
**Iron uptake**	fecI	BacPipe gbk file
Stress survival		
**KatA**	katA	BacPipe/VirDB
Regulation		
**BfmRS**	bfmR	ABRicate/VFDB
	bfmS	ABRicate/VFDB

Table S3: Metadata of the 433 isolates from Pathogenwatch

**Sample**	**Collection**	**Isolation source**	**Study accession**	**Country**	**ST-Pasteur**
SAMN15816307	2013	abdominal	PRJNA657148	Sweden	1
SAMN16541573	2010	abscessus	PRJNA671692	Hungary	1
SAMEA3724641	2008	bedsore	PRJEB12351	Germany	1

Sample	Collection	Isolation source	Study accession	Country	ST-Pasteur
SAMN20285909	2003	blood	PRJNA747293	France	1
SAMEA3724642	2008	blood culture	PRJEB12351	Germany	1
SAMEA7451180	2013	clinical material	PRJEB40828	Germany	1
SAMEA7451193	2015	clinical material	PRJEB40828	Germany	1
SAMEA7451222	2016	clinical material	PRJEB40828	Germany	1
SAMEA7451227	2017	clinical material	PRJEB40828	Germany	1
SAMEA7451237	2012	clinical material	PRJEB40828	Germany	1
SAMEA4916058	2017	Hospital	PRJEB28660	Switzerland	1
SAMN03160614	2013	Hospital	PRJNA266271	Denmark	1
SAMN08398972	2014	stool	PRJNA431710	Greece	1
SAMN08398946	2014	respiratory sample	PRJNA431710	Greece	1
SAMN08398930	2013	respiratory sample	PRJNA431710	Greece	1
SAMN08398928	2013	respiratory sample	PRJNA431710	Greece	1
SAMN08398948	2014	respiratory sample	PRJNA431710	Greece	1
SAMN08398947	2014	respiratory sample	PRJNA431710	Greece	1
SAMEA3724636	2008	tracheal secretion	PRJEB12351	Germany	1
SAMEA3724637	2008	tracheal secretion	PRJEB12351	Germany	1
SAMEA3724639	2008	tracheal secretion	PRJEB12351	Germany	1
SAMEA3724640	2008	tracheal secretion	PRJEB12351	Germany	1
SAMEA3724643	2008	tracheal secretion	PRJEB12351	Germany	1
SAMEA3724644	2008	tracheal secretion	PRJEB12351	Germany	1
SAMEA3724634	2008	tracheal secretion	PRJEB12351	Germany	1
SAMEA3724638	2008	tracheostoma	PRJEB12351	Germany	1
SAMN16541578	2010	urine	PRJNA671692	Hungary	1
SAMN01828150	1984	urine	PRJNA183259	Netherlands	1
SAMN01828143	1994	urine	PRJNA183252	Czech Republic	1
SAMN01828149	1994	sputum	PRJNA183258	Czech Republic	10
SAMN08398916	2013	respiratory sample	PRJNA431710	Italy	1066
SAMN08398922	2013	respiratory sample	PRJNA431710	Italy	1067
SAMN01828148	1994	Tracheal secretion	PRJNA183257	Czech Republic	11
SAMEA5396106	2013	perianal swab	PRJEB31555	Germany	113
SAMEA5396095	2013	rectal swab	PRJEB31555	Germany	113
SAMEA5396102	2013	tracheal secretion	PRJEB31555	Germany	113
SAMEA7451194	2015	clinical material	PRJEB40828	Germany	1142

Sample	Collection	Isolation source	Study accession	Country	ST-Pasteur
SAMN01828176	1994	Tracheal secretion	PRJNA183316	Czech Republic	12
SAMEA5229254	2018	clinical	PRJEB30466	Italy	1279
SAMEA5229255	2018	clinical	PRJEB30466	Italy	1280
SAMN25688258	2018	feces	PRJNA671692	Hungary	132
SAMEA2241593	2012	Urine	PRJEB4735	United Kingdom	136
SAMEA5226473	2018	screen	PRJEB30134	United Kingdom	141
SAMEA2241519	2012	CSF	PRJEB4735	United Kingdom	149
SAMEA8702129	2014	Blood stream	PRJEB44847	Sweden	15
SAMEA7451172	2013	clinical material	PRJEB40828	Germany	15
SAMEA7451244	2012	clinical material	PRJEB40828	Germany	15
SAMN09714556	2016	respiratory sample	PRJNA482774	Germany	15
SAMN01828174	2001	sputum	PRJNA183314	Czech Republic	15
SAMEA8702125	2014	Wound	PRJEB44847	Sweden	15
SAMEA8702126	2014	Wound	PRJEB44847	Sweden	15
SAMEA8702127	2014	Wound	PRJEB44847	Sweden	15
SAMEA8702128	2014	Wound	PRJEB44847	Sweden	15
SAMEA8702130	2015	Wound	PRJEB44847	Sweden	15
SAMEA8702131	2015	Wound	PRJEB44847	Sweden	15
SAMEA8702132	2015	Wound	PRJEB44847	Sweden	15
SAMEA8702133	2015	Wound	PRJEB44847	Sweden	15
SAMN09714555	2016	wound swab	PRJNA482774	Germany	15
SAMN25688249	2017	feces	PRJNA671692	Hungary	1561
SAMN03160609	2014	Hospital	PRJNA266271	Denmark	158
SAMEA7451185	2014	clinical material	PRJEB40828	Germany	164
SAMEA7451187	2014	clinical material	PRJEB40828	Germany	164
SAMEA6657151	2018	Urine	PRJEB37711	Denmark	164
SAMEA7451182	2013	clinical material	PRJEB40828	Germany	187
SAMN08581091	2013	bronchial aspirate	PRJNA435581	Italy	187
SAMN08398951	2014	respiratory sample	PRJNA431710	Greece	187
SAMEA7451205	2016	clinical material	PRJEB40828	Germany	19

Sample	Collection	Isolation source	Study accession	Country	ST-Pasteur
SAMEA2241595	2012	Tissue	PRJEB4735	United Kingdom	195
SAMEA2241594	2012	Tissue	PRJEB4735	United Kingdom	195
SAMEA2241596	2012	Sputum	PRJEB4735	United Kingdom	195
SAMEA2241584	2012	Sputum	PRJEB4735	United Kingdom	195
SAMN08159980	2017	abdominal dreinage	PRJNA427128	Italy	2
SAMEA6451095	2018	biliary fluid	PRJEB30134	United Kingdom	2
SAMEA5226480	2018	blood	PRJEB30134	United Kingdom	2
SAMN16541575	2010	blood	PRJNA671692	Hungary	2
SAMN08159985	2017	blood culture	PRJNA427128	Italy	2
SAMN08159987	2017	blood culture	PRJNA427128	Italy	2
SAMN08159989	2017	blood culture	PRJNA427128	Italy	2
SAMN08159979	2017	blood culture	PRJNA427128	Italy	2
SAMN08159977	2017	blood culture	PRJNA427128	Italy	2
SAMN08159978	2017	blood culture	PRJNA427128	Italy	2
SAMN09202765	2017	Hemoculture	PRJNA387062	Romania	2
SAMN09202766	2018	Hemoculture	PRJNA387062	Romania	2
SAMEA5396116	2012	catheter swab	PRJEB31555	Germany	2
SAMEA5396112	2014	catheter urine	PRJEB31555	Germany	2
SAMN01828175	2003	I.V. Cannula	PRJNA183315	Czech Republic	2
SAMEA5229256	2018	clinical	PRJEB30466	Italy	2
SAMEA5396094	2012	clinical material	PRJEB31555	Germany	2
SAMEA7451168	2012	clinical material	PRJEB40828	Germany	2
SAMEA7451169	2012	clinical material	PRJEB40828	Germany	2
SAMEA7451170	2012	clinical material	PRJEB40828	Germany	2
SAMEA7451171	2013	clinical material	PRJEB40828	Germany	2
SAMEA7451175	2013	clinical material	PRJEB40828	Germany	2
SAMEA7451176	2013	clinical material	PRJEB40828	Germany	2
SAMEA7451179	2013	clinical material	PRJEB40828	Germany	2
SAMEA7451181	2013	clinical material	PRJEB40828	Germany	2

Sample	Collection	Isolation source	Study accession	Country	ST-Pasteur
SAMEA7451183	2014	clinical material	PRJEB40828	Germany	2
SAMEA7451186	2014	clinical material	PRJEB40828	Germany	2
SAMEA7451188	2014	clinical material	PRJEB40828	Germany	2
SAMEA7451189	2014	clinical material	PRJEB40828	Germany	2
SAMEA7451190	2015	clinical material	PRJEB40828	Germany	2
SAMEA7451191	2015	clinical material	PRJEB40828	Germany	2
SAMEA7451192	2015	clinical material	PRJEB40828	Germany	2
SAMEA7451196	2015	clinical material	PRJEB40828	Germany	2
SAMEA7451197	2015	clinical material	PRJEB40828	Germany	2
SAMEA7451199	2015	clinical material	PRJEB40828	Germany	2
SAMEA7451200	2015	clinical material	PRJEB40828	Germany	2
SAMEA7451201	2015	clinical material	PRJEB40828	Germany	2
SAMEA7451202	2015	clinical material	PRJEB40828	Germany	2
SAMEA7451203	2016	clinical material	PRJEB40828	Germany	2
SAMEA7451204	2016	clinical material	PRJEB40828	Germany	2
SAMEA7451206	2016	clinical material	PRJEB40828	Germany	2
SAMEA7451207	2016	clinical material	PRJEB40828	Germany	2
SAMEA7451208	2016	clinical material	PRJEB40828	Germany	2
SAMEA7451209	2015	clinical material	PRJEB40828	Germany	2
SAMEA7451210	2015	clinical material	PRJEB40828	Germany	2
SAMEA7451211	2014	clinical material	PRJEB40828	Germany	2
SAMEA7451212	2015	clinical material	PRJEB40828	Germany	2
SAMEA7451213	2015	clinical material	PRJEB40828	Germany	2
SAMEA7451215	2015	clinical material	PRJEB40828	Germany	2
SAMEA7451216	2016	clinical material	PRJEB40828	Germany	2
SAMEA7451217	2016	clinical material	PRJEB40828	Germany	2
SAMEA7451218	2016	clinical material	PRJEB40828	Germany	2
SAMEA7451220	2016	clinical material	PRJEB40828	Germany	2
SAMEA7451221	2016	clinical material	PRJEB40828	Germany	2
SAMEA7451224	2017	clinical material	PRJEB40828	Germany	2
SAMEA7451225	2017	clinical material	PRJEB40828	Germany	2
SAMEA7451228	2017	clinical material	PRJEB40828	Germany	2
SAMEA7451229	2017	clinical material	PRJEB40828	Germany	2
SAMEA7451233	2017	clinical material	PRJEB40828	Germany	2

Sample	Collection	Isolation source	Study accession	Country	ST-Pasteur
SAMEA7451234	2017	clinical material	PRJEB40828	Germany	2
SAMEA7451236	2012	clinical material	PRJEB40828	Germany	2
SAMEA7451238	2018	clinical material	PRJEB40828	Germany	2
SAMEA7451239	2015	clinical material	PRJEB40828	Germany	2
SAMEA7451240	2015	clinical material	PRJEB40828	Germany	2
SAMEA7451241	2012	clinical material	PRJEB40828	Germany	2
SAMEA7451247	2018	clinical material	PRJEB40828	Germany	2
SAMEA4916047	2017	Hospital	PRJEB28660	Switzerland	2
SAMEA4916062	2017	Hospital	PRJEB28660	Switzerland	2
SAMEA4916067	2017	Hospital	PRJEB28660	Switzerland	2
SAMEA9459703	2019	Hospital	PRJEB46126	Netherlands	2
SAMEA9459702	2019	Hospital	PRJEB46126	Netherlands	2
SAMN03160611	2013	Hospital	PRJNA266271	Denmark	2
SAMN03160612	2013	Hospital	PRJNA266271	Denmark	2
SAMN03160613	2013	Hospital	PRJNA266271	Denmark	2
SAMN03160616	2013	Hospital	PRJNA266271	Denmark	2
SAMN03160617	2013	Hospital	PRJNA266271	Denmark	2
SAMEA5226470	2018	screen	PRJEB30134	United Kingdom	2
SAMEA6451101	2018	screen	PRJEB30134	United Kingdom	2
SAMEA5396122	2012	screening swab	PRJEB31555	Germany	2
SAMEA2241521	2012	Tissue	PRJEB4735	United Kingdom	2
SAMN09302593	2005	cerebrospinal fluid	PRJNA474045	Italy	2
SAMN03577729	2013	Decubitus pressure sore	PRJNA282640	Sweden	2
SAMEA5396120	2013	drainage liquid	PRJEB31555	Germany	2
SAMEA5789902	2016	Stool	PRJEB34513	United Kingdom	2
SAMN08398970	2013	stool	PRJNA431710	Spain	2
SAMN08398971	2013	stool	PRJNA431710	Spain	2
SAMN09714553	na	stool	PRJNA482774	Spain	2
SAMN09714554	2012	stool	PRJNA482774	Spain	2
SAMN17915434	2010	fistula	PRJNA701882	Poland	2

Sample	Collection	Isolation source	Study accession	Country	ST-Pasteur
SAMEA5396113	2014	groin swab	PRJEB31555	Germany	2
SAMN14605985	2017	Human sample	PRJNA625727	Germany	2
SAMN14605984	na	Human sample	PRJNA625727	Finland	2
SAMN14605983	na	Human sample	PRJNA625727	Finland	2
SAMN14605982	na	Human sample	PRJNA625727	Finland	2
SAMEA5396087	2013	rectal swab	PRJEB31555	Germany	2
SAMEA5396088	2013	rectal swab	PRJEB31555	Germany	2
SAMEA5396090	2015	rectal swab	PRJEB31555	Germany	2
SAMEA5396092	2015	rectal swab	PRJEB31555	Germany	2
SAMEA5396100	2015	rectal swab	PRJEB31555	Germany	2
SAMEA5396108	2014	rectal swab	PRJEB31555	Germany	2
SAMEA5396110	2013	rectal swab	PRJEB31555	Germany	2
SAMN17915442	2012	rectal swab	PRJNA701882	Poland	2
SAMN08159986	2017	rectal swab	PRJNA427128	Italy	2
SAMN08159983	2017	rectal swab	PRJNA427128	Italy	2
SAMN08159981	2017	rectal swab	PRJNA427128	Italy	2
SAMN08159976	2017	rectal swab	PRJNA427128	Italy	2
SAMN16541582	2017	bronchial	PRJNA671692	Hungary	2
SAMN16541580	2010	bronchial	PRJNA671692	Hungary	2
SAMN16541579	2010	bronchial	PRJNA671692	Hungary	2
SAMN16541586	2017	bronchial	PRJNA671692	Hungary	2
SAMN08581100	2013	bronchial aspirate	PRJNA435581	Italy	2
SAMN08581099	2013	bronchial aspirate	PRJNA435581	Italy	2
SAMN08581096	2013	bronchial aspirate	PRJNA435581	Italy	2
SAMN08581095	2013	bronchial aspirate	PRJNA435581	Italy	2
SAMN08581088	2013	bronchial aspirate	PRJNA435581	Italy	2
SAMN08581087	2013	bronchial aspirate	PRJNA435581	Italy	2
SAMEA5396117	2014	bronchial secretion	PRJEB31555	Germany	2
SAMEA5396124	2013	bronchial secretion	PRJEB31555	Germany	2
SAMN17915440	2012	bronchial secretion	PRJNA701882	Poland	2
SAMN17915445	2013	bronchial secretion	PRJNA701882	Poland	2
SAMN17915435	2010	bronchial secretion	PRJNA701882	Poland	2
SAMN08159988	2017	broncho alveolar fluid	PRJNA427128	Italy	2
SAMN08159984	2017	broncho alveolar fluid	PRJNA427128	Italy	2

Sample	Collection	Isolation source	Study accession	Country	ST-Pasteur
SAMN08159982	2017	broncho alveolar fluid	PRJNA427128	Italy	2
SAMN08398911	2013	respiratory sample	PRJNA431710	Spain	2
SAMN08398912	2013	respiratory sample	PRJNA431710	Spain	2
SAMN08398913	2013	respiratory sample	PRJNA431710	Spain	2
SAMN08398914	2013	respiratory sample	PRJNA431710	Italy	2
SAMN08398915	2013	respiratory sample	PRJNA431710	Italy	2
SAMN08398917	2013	respiratory sample	PRJNA431710	Greece	2
SAMN08398918	2013	respiratory sample	PRJNA431710	Greece	2
SAMN08398919	2013	respiratory sample	PRJNA431710	Greece	2
SAMN08398920	2013	respiratory sample	PRJNA431710	Greece	2
SAMN08398909	2012	respiratory sample	PRJNA431710	Spain	2
SAMN08398964	2015	respiratory sample	PRJNA431710	Greece	2
SAMN08398960	2015	respiratory sample	PRJNA431710	Greece	2
SAMN08398966	2015	respiratory sample	PRJNA431710	Greece	2
SAMN08398962	2014	respiratory sample	PRJNA431710	Greece	2
SAMN08398942	2014	respiratory sample	PRJNA431710	Greece	2
SAMN08398957	2014	respiratory sample	PRJNA431710	Greece	2
SAMN08398950	2014	respiratory sample	PRJNA431710	Italy	2
SAMN08398949	2014	respiratory sample	PRJNA431710	Greece	2
SAMN08398929	2013	respiratory sample	PRJNA431710	Greece	2
SAMN08398963	2015	respiratory sample	PRJNA431710	Greece	2
SAMN08398967	2015	respiratory sample	PRJNA431710	Greece	2
SAMN08398926	2013	respiratory sample	PRJNA431710	Greece	2
SAMN08398925	2013	respiratory sample	PRJNA431710	Greece	2
SAMN08398927	2013	respiratory sample	PRJNA431710	Greece	2
SAMN08398921	2013	respiratory sample	PRJNA431710	Spain	2
SAMN08398924	2013	respiratory sample	PRJNA431710	Greece	2
SAMN08398923	2013	respiratory sample	PRJNA431710	Italy	2
SAMN08398959	2015	respiratory sample	PRJNA431710	Italy	2
SAMN08398965	2015	respiratory sample	PRJNA431710	Greece	2
SAMN08398945	2014	respiratory sample	PRJNA431710	Greece	2
SAMN08398953	2014	respiratory sample	PRJNA431710	Italy	2
SAMN08398941	2014	respiratory sample	PRJNA431710	Greece	2
SAMN08398954	2014	respiratory sample	PRJNA431710	Italy	2

Sample	Collection	Isolation source	Study accession	Country	ST-Pasteur
SAMN08398944	2014	respiratory sample	PRJNA431710	Greece	2
SAMN08398943	2014	respiratory sample	PRJNA431710	Greece	2
SAMN08398906	2012	respiratory sample	PRJNA431710	Spain	2
SAMN08398910	2013	respiratory sample	PRJNA431710	Spain	2
SAMN08398955	2014	respiratory sample	PRJNA431710	Greece	2
SAMN08398956	2014	respiratory sample	PRJNA431710	Spain	2
SAMN08398958	2015	respiratory sample	PRJNA431710	Italy	2
SAMN08398952	2014	respiratory sample	PRJNA431710	Greece	2
SAMN08398939	2014	respiratory sample	PRJNA431710	Greece	2
SAMN08398940	2014	respiratory sample	PRJNA431710	Greece	2
SAMN08398937	2014	respiratory sample	PRJNA431710	Italy	2
SAMN08398938	2013	respiratory sample	PRJNA431710	Greece	2
SAMN08398935	2014	respiratory sample	PRJNA431710	Italy	2
SAMN08398936	2014	respiratory sample	PRJNA431710	Italy	2
SAMN08398933	2014	respiratory sample	PRJNA431710	Greece	2
SAMN08398934	2014	respiratory sample	PRJNA431710	Spain	2
SAMN08398931	2013	respiratory sample	PRJNA431710	Greece	2
SAMN08398932	2013	respiratory sample	PRJNA431710	Greece	2
SAMEA5396098	2014	tracheal secretion	PRJEB31555	Germany	2
SAMEA5396103	2013	tracheal secretion	PRJEB31555	Germany	2
SAMEA5396104	2013	tracheal secretion	PRJEB31555	Germany	2
SAMEA5396109	2012	tracheal secretion	PRJEB31555	Germany	2
SAMEA5396119	2013	tracheal secretion	PRJEB31555	Germany	2
SAMEA5396121	2013	tracheal secretion	PRJEB31555	Germany	2
SAMEA5226469	2018	Sputum	PRJEB30134	United Kingdom	2
SAMN16541585	2017	sputum	PRJNA671692	Hungary	2
SAMN16541583	2017	sputum	PRJNA671692	Hungary	2
SAMEA5396115	2014	stoma swab	PRJEB31555	Germany	2
SAMN17915443	2014	urine	PRJNA701882	Poland	2
SAMN17915441	2012	urine	PRJNA701882	Poland	2
SAMN17915439	2012	urine	PRJNA701882	Poland	2
SAMN17915438	2012	urine	PRJNA701882	Poland	2
SAMN17915446	2013	urine	PRJNA701882	Poland	2

Sample	Collection	Isolation source	Study accession	Country	ST-Pasteur
SAMN17915444	2013	urine	PRJNA701882	Poland	2
SAMN01828145	1982	urine	PRJNA183254	Netherlands	2
SAMN01087912	1991	urine	PRJNA183334	Czech Republic	2
SAMN07602915	2012	wound	PRJNA401330	Sweden	2
SAMEA5396097	2012	wound swab	PRJEB31555	Germany	2
SAMEA5396099	2012	wound swab	PRJEB31555	Germany	2
SAMEA5396105	2013	wound swab	PRJEB31555	Germany	2
SAMEA5396114	2015	wound swab	PRJEB31555	Germany	2
SAMEA5396118	2012	wound swab	PRJEB31555	Germany	2
SAMEA2241526	2011	Wound swab	PRJEB4735	United Kingdom	2
SAMEA2241586	2011	Wound swab	PRJEB4735	United Kingdom	2
SAMN17915437	2012	wound swab	PRJNA701882	Poland	2
SAMN17915436	2011	wound swab	PRJNA701882	Poland	2
SAMN17915447	2013	wound swab	PRJNA701882	Poland	2
SAMEA5226451	2017	screen	PRJEB30134	United Kingdom	203
SAMN03576449	2013	blood	PRJNA282618	Sweden	25
SAMN03366519	2000	respiratory tract	PRJNA276040	Netherlands	25
SAMN03366525	2007	respiratory tract	PRJNA276040	Germany	25
SAMN11458717	2017	sputum	PRJNA269675	Russia	25
SAMN03366515	1985	Umbilicus	PRJNA276040	Netherlands	25
SAMN01828177	1993	wound	PRJNA183317	Czech Republic	25
SAMEA6451151	2017	screen	PRJEB30134	United Kingdom	273
SAMN01828181	1997	blood	PRJNA183321	Netherlands	3
SAMEA5396089	2013	clinical material	PRJEB31555	Germany	315
SAMN01828146	1992	Tracheal secretion	PRJNA183255	Czech Republic	35
SAMN01828144	1992	Tracheal secretion	PRJNA183253	Czech Republic	36
SAMN01828142	1993	I.V. Cannula	PRJNA183251	Czech Republic	37
SAMN01828151	1992	nasal	PRJNA183260	Czech Republic	38
SAMN01828139	1996	Cannula	PRJNA183248	Czech Republic	39
SAMN01828147	1993	urine	PRJNA183256	Czech Republic	40

Sample	Collection	Isolation source	Study accession	Country	ST-Pasteur
SAMEA7451230	2017	clinical material	PRJEB40828	Germany	400
SAMEA7451231	2018	clinical material	PRJEB40828	Germany	400
SAMEA7451235	2018	clinical material	PRJEB40828	Germany	400
SAMEA5396107	2015	wound swab	PRJEB31555	Germany	400
SAMN03366522	2002	venous catheter tip	PRJNA276040	Netherlands	402
SAMN20286174	1966	ear pus	PRJNA747293	France	411
SAMN05730797	1951	patient	PRJNA341952	France	437
SAMN05730819	1951	patient	PRJNA341952	France	437
SAMN05730834	1951	patient	PRJNA341952	France	437
SAMN05730835	1951	patient	PRJNA341952	France	437
SAMN16541577	2010	canule	PRJNA671692	Hungary	45
SAMEA7451178	2013	clinical material	PRJEB40828	Germany	45
SAMEA7451198	2015	clinical material	PRJEB40828	Germany	45
SAMN25688248	2018	feces	PRJNA671692	Hungary	45
SAMN16541576	2010	bronchial	PRJNA671692	Hungary	45
SAMN01828180	2000	tracheal aspirate	PRJNA183320	Czech Republic	47
SAMN09202764	2017	Catheter	PRJNA387062	Romania	492
SAMN25688252	2017	feces	PRJNA671692	Hungary	492
SAMN25688251	2018	feces	PRJNA671692	Hungary	492
SAMN16541584	2017	bronchial	PRJNA671692	Hungary	492
SAMN16541588	2017	trachea	PRJNA671692	Hungary	492
SAMEA4780633	2017	Human	PRJEB27660	Germany	494
SAMEA4780635	2017	Human	PRJEB27660	Germany	494
SAMEA4780636	2017	Human	PRJEB27660	Germany	494
SAMEA4780637	2017	Human	PRJEB27660	Germany	494
SAMEA4780640	2017	Human	PRJEB27660	Germany	494
SAMEA7451243	2014	clinical material	PRJEB40828	Germany	499
SAMN08398907	2012	respiratory sample	PRJNA431710	Spain	537
SAMN08398908	2012	respiratory sample	PRJNA431710	Spain	537
SAMEA7451195	2015	clinical material	PRJEB40828	Germany	570
SAMEA7451232	2018	clinical material	PRJEB40828	Germany	571
SAMEA7451226	2017	clinical material	PRJEB40828	Germany	604
SAMEA4780634	2017	Human	PRJEB27660	Germany	2527
SAMEA4780638	2017	Human	PRJEB27660	Germany	2527

Sample	Collection	Isolation source	Study accession	Country	ST-Pasteur
SAMEA4780639	2017	Human	PRJEB27660	Germany	2527
SAMEA4780641	2017	Human	PRJEB27660	Germany	2527
SAMEA4780642	2017	Human	PRJEB27660	Germany	2527
SAMEA4780643	2017	Human	PRJEB27660	Germany	2527
SAMEA4780644	2017	Human	PRJEB27660	Germany	2527
SAMN08398961	2015	respiratory sample	PRJNA431710	Italy	632
SAMN09202763	2017	Cerebrospinal fluid	PRJNA387062	Romania	636
SAMN16541594	2017	drain	PRJNA671692	Hungary	636
SAMN25688257	2018	feces	PRJNA671692	Hungary	636
SAMN25688256	2017	feces	PRJNA671692	Hungary	636
SAMN25688255	2017	feces	PRJNA671692	Hungary	636
SAMN25688254	2017	feces	PRJNA671692	Hungary	636
SAMN25688253	2017	feces	PRJNA671692	Hungary	636
SAMN25688250	2019	feces	PRJNA671692	Hungary	636
SAMN25688263	2019	feces	PRJNA671692	Hungary	636
SAMN25688262	2017	feces	PRJNA671692	Hungary	636
SAMN25688261	2017	feces	PRJNA671692	Hungary	636
SAMN25688260	2017	feces	PRJNA671692	Hungary	636
SAMN25688259	2018	feces	PRJNA671692	Hungary	636
SAMN16541592	2017	nasal	PRJNA671692	Hungary	636
SAMN16541595	2017	bronchial	PRJNA671692	Hungary	636
SAMN16541591	2017	bronchial	PRJNA671692	Hungary	636
SAMN16541590	2017	bronchial	PRJNA671692	Hungary	636
SAMN16541589	2017	bronchial	PRJNA671692	Hungary	636
SAMN16541587	2017	bronchial	PRJNA671692	Hungary	636
SAMN03577730	2013	Bronchial secretion	PRJNA282641	Sweden	636
SAMN16541593	2017	throat	PRJNA671692	Hungary	636
SAMN16541574	2010	throat	PRJNA671692	Hungary	636
SAMEA5396091	2015	wound swab	PRJEB31555	Germany	636
SAMEA6451152	2017	screen	PRJEB30134	United Kingdom	664
SAMEA5396111	2015	clinical material	PRJEB31555	Germany	717
SAMN08398969	2012	stool	PRJNA431710	Spain	745
SAMN08398968	2012	stool	PRJNA431710	Spain	745

Sample	Collection	Isolation source	Study accession	Country	ST-Pasteur
SAMN09714552	2012	stool	PRJNA482774	Spain	745
SAMN08398905	2012	respiratory sample	PRJNA431710	Spain	745
SAMN08398901	2012	respiratory sample	PRJNA431710	Spain	745
SAMN08398902	2012	respiratory sample	PRJNA431710	Spain	745
SAMN08398903	2012	respiratory sample	PRJNA431710	Spain	745
SAMN08398904	2012	respiratory sample	PRJNA431710	Spain	745
SAMEA7451173	2013	clinical material	PRJEB40828	Germany	78
SAMEA7451184	2014	clinical material	PRJEB40828	Germany	78
SAMEA7451242	2012	clinical material	PRJEB40828	Germany	78
SAMEA7451245	2018	clinical material	PRJEB40828	Germany	78
SAMEA4916054	2017	Hospital	PRJEB28660	Switzerland	78
SAMEA4916094	2010	Hospital	PRJEB28660	Switzerland	78
SAMN20110676	2012	urinary	PRJNA744549	Germany	78
SAMN16541581	2010	blood	PRJNA671692	Hungary	79
SAMEA7451219	2016	clinical material	PRJEB40828	Germany	81
SAMEA2241583	2011	Burn swab	PRJEB4735	United Kingdom	821
SAMEA7451174	2013	clinical material	PRJEB40828	Germany	Novel ST: *7541
SAMEA7451177	2013	clinical material	PRJEB40828	Germany	Novel ST: *7541
SAMN15676490	2019	clinical	PRJNA649662	Spain	85
SAMEA5396101	2015	wound swab	PRJEB31555	Germany	85
SAMEA2241279	2012	Abdo Drain	PRJEB4735	United Kingdom	94
SAMEA2241471	2013	Anaesthetic machine Burns theatre	PRJEB4735	United Kingdom	94
SAMEA2241592	2012	B/C	PRJEB4735	United Kingdom	94
SAMEA2241506	2012	Blood	PRJEB4735	United Kingdom	94
SAMEA2241277	2011	Burn Swab	PRJEB4735	United Kingdom	94
SAMEA2241505	2012	Burn Swab	PRJEB4735	United Kingdom	94

Sample	Collection	Isolation source	Study accession	Country	ST-Pasteur
SAMEA2241507	2012	Burn Swab	PRJEB4735	United Kingdom	94
SAMEA2241412	2012	Burn Swab	PRJEB4735	United Kingdom	94
SAMEA2241413	2012	Burn Swab	PRJEB4735	United Kingdom	94
SAMEA2241393	2011	Burn Swab	PRJEB4735	United Kingdom	94
SAMEA2241591	2012	Burn swab	PRJEB4735	United Kingdom	94
SAMEA2241598	2012	Burn swab	PRJEB4735	United Kingdom	94
SAMEA2241601	2012	Burn swab	PRJEB4735	United Kingdom	94
SAMEA2241469	2012	Burn swab	PRJEB4735	United Kingdom	94
SAMEA2241603	2012	Burn swab	PRJEB4735	United Kingdom	94
SAMEA2241479	2012	Burn swab	PRJEB4735	United Kingdom	94
SAMEA2241488	2011	Burn swab	PRJEB4735	United Kingdom	94
SAMEA2241582	2013	Burn swab	PRJEB4735	United Kingdom	94
SAMEA2241489	2011	Burn swab	PRJEB4735	United Kingdom	94
SAMEA2241578	2013	Burn swab	PRJEB4735	United Kingdom	94
SAMEA2241581	2013	Burn swab	PRJEB4735	United Kingdom	94
SAMEA2241577	2013	Central venous Catheter tip	PRJEB4735	United Kingdom	94
SAMEA2241585	2006	Chest drain	PRJEB4735	United Kingdom	94
SAMEA2241407	2012	CSF	PRJEB4735	United Kingdom	94

Sample	Collection	Isolation source	Study accession	Country	ST-Pasteur
SAMEA2241282	2012	CSF	PRJEB4735	United Kingdom	94
SAMEA2241406	2012	Lumbar Drain	PRJEB4735	United Kingdom	94
SAMEA2241522	na	Original	PRJEB4735	United Kingdom	94
SAMEA2241411	2012	Site Swab	PRJEB4735	United Kingdom	94
SAMEA2241510	2012	Site Swab	PRJEB4735	United Kingdom	94
SAMEA2241516	2012	Site swab	PRJEB4735	United Kingdom	94
SAMEA2241572	2012	Site swab	PRJEB4735	United Kingdom	94
SAMEA2241604	2012	Pus swab, LBurn swab testicle	PRJEB4735	United Kingdom	94
SAMEA2241278	2011	BAL fluid	PRJEB4735	United Kingdom	94
SAMEA2241280	2012	Sputum	PRJEB4735	United Kingdom	94
SAMEA2241405	2012	Sputum	PRJEB4735	United Kingdom	94
SAMEA2241409	2012	Sputum	PRJEB4735	United Kingdom	94
SAMEA2241392	2011	Sputum	PRJEB4735	United Kingdom	94
SAMEA2241512	2012	Sputum	PRJEB4735	United Kingdom	94
SAMEA2241883	2012	Sputum	PRJEB4735	United Kingdom	94
SAMEA2241486	2011	Sputum	PRJEB4735	United Kingdom	94
SAMEA2241576	2013	Sputum	PRJEB4735	United Kingdom	94
SAMEA2241487	2011	Sputum	PRJEB4735	United Kingdom	94

Sample	Collection	Isolation source	Study accession	Country	ST-Pasteur
SAMEA2241573	2012	Sputum	PRJEB4735	United Kingdom	94
SAMEA2241574	2012	Sputum	PRJEB4735	United Kingdom	94
SAMEA2241575	2012	Sputum	PRJEB4735	United Kingdom	94
SAMEA2241579	2013	Sputum	PRJEB4735	United Kingdom	94
SAMEA2241509	2012	Wound Swab	PRJEB4735	United Kingdom	94
SAMEA2241511	2012	Wound Swab	PRJEB4735	United Kingdom	94
SAMEA2241397	2011	Wound Swab	PRJEB4735	United Kingdom	94
SAMEA2241398	2012	Wound Swab	PRJEB4735	United Kingdom	94
SAMEA2241402	2012	Wound Swab	PRJEB4735	United Kingdom	94
SAMEA2241283	2012	Wound Swab	PRJEB4735	United Kingdom	94
SAMEA2241599	2012	Wound swab	PRJEB4735	United Kingdom	94
SAMEA2241605	2012	Wound swab	PRJEB4735	United Kingdom	94
SAMEA2241580	2013	Wound swab	PRJEB4735	United Kingdom	94

Table S4: Metadata of the 176 ST2 isolates from Pathogenwatch

Sample accession	Collection year	Isolation source	Study accession	Country	ST-Pasteur
SAMEA5396092	2015	rectal swab	PRJEB31555	Germany	2
SAMEA7451224	2017	clinical material	PRJEB40828	Germany	2
SAMEA7451225	2017	clinical material	PRJEB40828	Germany	2
SAMEA2241521	2012	Tissue	PRJEB4735	United Kingdom	2
SAMN08398911	2013	respiratory sample	PRJNA431710	Spain	2
SAMN08398906	2012	respiratory sample	PRJNA431710	Spain	2
SAMN09714554	2012	stool	PRJNA482774	Spain	2
SAMN14605984	na	Human sample	PRJNA625727	Finland	2
SAMN14605982	na	Human sample	PRJNA625727	Finland	2
SAMEA5226480	2018	blood	PRJEB30134	United Kingdom	2
SAMEA4916047	2017	Hospital	PRJEB28660	Switzerland	2
SAMN17915442	2012	rectal swab	PRJNA701882	Poland	2
SAMN17915441	2012	urine	PRJNA701882	Poland	2
SAMN17915446	2013	urine	PRJNA701882	Poland	2
SAMEA7451216	2016	clinical material	PRJEB40828	Germany	2
SAMEA7451169	2012	clinical material	PRJEB40828	Germany	2
SAMEA7451196	2015	clinical material	PRJEB40828	Germany	2
SAMEA7451197	2015	clinical material	PRJEB40828	Germany	2
SAMEA7451200	2015	clinical material	PRJEB40828	Germany	2
SAMEA7451201	2015	clinical material	PRJEB40828	Germany	2
SAMEA7451202	2015	clinical material	PRJEB40828	Germany	2
SAMEA7451203	2016	clinical material	PRJEB40828	Germany	2
SAMEA7451204	2016	clinical material	PRJEB40828	Germany	2
SAMEA7451206	2016	clinical material	PRJEB40828	Germany	2
SAMEA7451207	2016	clinical material	PRJEB40828	Germany	2
SAMEA7451208	2016	clinical material	PRJEB40828	Germany	2
SAMEA7451213	2015	clinical material	PRJEB40828	Germany	2
SAMEA7451217	2016	clinical material	PRJEB40828	Germany	2

SAMN08159979	2017	blood culture	PRJNA427128	Italy	2
SAMN08398959	2015	respiratory sample	PRJNA431710	Italy	2
SAMN08398936	2014	respiratory sample	PRJNA431710	Italy	2
SAMN08581088	2013	bronchial aspirate	PRJNA435581	Italy	2
SAMN08581087	2013	bronchial aspirate	PRJNA435581	Italy	2
SAMN17915445	2013	bronchial secretion	PRJNA701882	Poland	2
SAMN17915444	2013	urine	PRJNA701882	Poland	2
SAMN17915437	2012	wound swab	PRJNA701882	Poland	2
SAMN17915436	2011	wound swab	PRJNA701882	Poland	2
SAMN17915447	2013	wound swab	PRJNA701882	Poland	2
SAMEA5396108	2014	rectal swab	PRJEB31555	Germany	2
SAMN08398970	2013	stool	PRJNA431710	Spain	2
SAMN08398965	2015	respiratory sample	PRJNA431710	Greece	2
SAMN08398955	2014	respiratory sample	PRJNA431710	Greece	2
SAMN08398935	2014	respiratory sample	PRJNA431710	Italy	2
SAMEA7451210	2015	clinical material	PRJEB40828	Germany	2
SAMN08398929	2013	respiratory sample	PRJNA431710	Greece	2
SAMN09202765	2017	Hemoculture	PRJNA387062	Romania	2
SAMEA5229256	2018	clinical	PRJEB30466	Italy	2
SAMEA5396113	2014	groin swab	PRJEB31555	Germany	2
SAMEA5226470	2018	screen	PRJEB30134	United Kingdom	2
SAMN09302593	2005	cerebrospinal fluid	PRJNA474045	Italy	2
SAMEA5396114	2015	wound swab	PRJEB31555	Germany	2
SAMEA4916062	2017	Hospital	PRJEB28660	Switzerland	2
SAMEA4916067	2017	Hospital	PRJEB28660	Switzerland	2
SAMEA5396094	2012	clinical material	PRJEB31555	Germany	2
SAMEA5396087	2013	rectal swab	PRJEB31555	Germany	2
SAMEA5396088	2013	rectal swab	PRJEB31555	Germany	2
SAMEA5396090	2015	rectal swab	PRJEB31555	Germany	2
SAMEA5396098	2014	tracheal secretion	PRJEB31555	Germany	2
SAMEA5789902	2016	Stool	PRJEB34513	United Kingdom	2
SAMEA7451170	2012	clinical material	PRJEB40828	Germany	2
SAMEA7451183	2014	clinical material	PRJEB40828	Germany	2

SAMEA7451186	2014	clinical material	PRJEB40828	Germany	2
SAMEA7451188	2014	clinical material	PRJEB40828	Germany	2
SAMEA7451199	2015	clinical material	PRJEB40828	Germany	2
SAMEA7451209	2015	clinical material	PRJEB40828	Germany	2
SAMEA7451212	2015	clinical material	PRJEB40828	Germany	2
SAMEA7451215	2015	clinical material	PRJEB40828	Germany	2
SAMEA7451218	2016	clinical material	PRJEB40828	Germany	2
SAMEA7451234	2017	clinical material	PRJEB40828	Germany	2
SAMEA7451247	2018	clinical material	PRJEB40828	Germany	2
SAMN03160616	2013	Hospital	PRJNA266271	Denmark	2
SAMN03160617	2013	Hospital	PRJNA266271	Denmark	2
SAMN09202766	2018	Hemoculture	PRJNA387062	Romania	2
SAMN08398967	2015	respiratory sample	PRJNA431710	Greece	2
SAMN14605985	2017	Human sample	PRJNA625727	Germany	2
SAMN17915440	2012	bronchial secretion	PRJNA701882	Poland	2
SAMN17915443	2014	urine	PRJNA701882	Poland	2
SAMN08398964	2015	respiratory sample	PRJNA431710	Greece	2
SAMN08398960	2015	respiratory sample	PRJNA431710	Greece	2
SAMN08398966	2015	respiratory sample	PRJNA431710	Greece	2
SAMN08398962	2014	respiratory sample	PRJNA431710	Greece	2
SAMN08398953	2014	respiratory sample	PRJNA431710	Italy	2
SAMN08398954	2014	respiratory sample	PRJNA431710	Italy	2
SAMN08398956	2014	respiratory sample	PRJNA431710	Spain	2
SAMN17915439	2012	urine	PRJNA701882	Poland	2
SAMN17915438	2012	urine	PRJNA701882	Poland	2
SAMEA7451175	2013	clinical material	PRJEB40828	Germany	2
SAMEA7451176	2013	clinical material	PRJEB40828	Germany	2
SAMEA7451179	2013	clinical material	PRJEB40828	Germany	2
SAMEA7451233	2017	clinical material	PRJEB40828	Germany	2
SAMEA7451241	2012	clinical material	PRJEB40828	Germany	2
SAMEA2241526	2011	Wound swab	PRJEB4735	United Kingdom	2
SAMEA2241586	2011	Wound swab	PRJEB4735	United Kingdom	2
SAMEA7451190	2015	clinical material	PRJEB40828	Germany	2

SAMEA7451191	2015	clinical material	PRJEB40828	Germany	2
SAMEA7451192	2015	clinical material	PRJEB40828	Germany	2
SAMEA7451238	2018	clinical material	PRJEB40828	Germany	2
SAMN01828145	1982	urine	PRJNA183254	Netherlands	2
SAMEA5396110	2013	rectal swab	PRJEB31555	Germany	2
SAMEA7451236	2012	clinical material	PRJEB40828	Germany	2
SAMN07602915	2012	wound	PRJNA401330	Sweden	2
SAMEA5396099	2012	wound swab	PRJEB31555	Germany	2
SAMEA5396112	2014	catheter urine	PRJEB31555	Germany	2
SAMEA7451229	2017	clinical material	PRJEB40828	Germany	2
SAMEA9459702	2019	Hospital	PRJEB46126	Netherlands	2
SAMEA7451168	2012	clinical material	PRJEB40828	Germany	2
SAMEA5396100	2015	rectal swab	PRJEB31555	Germany	2
SAMEA6451095	2018	biliary fluid	PRJEB30134	United Kingdom	2
SAMEA5396103	2013	tracheal secretion	PRJEB31555	Germany	2
SAMEA5396104	2013	tracheal secretion	PRJEB31555	Germany	2
SAMEA7451171	2013	clinical material	PRJEB40828	Germany	2
SAMEA7451181	2013	clinical material	PRJEB40828	Germany	2
SAMEA7451189	2014	clinical material	PRJEB40828	Germany	2
SAMEA7451211	2014	clinical material	PRJEB40828	Germany	2
SAMEA7451220	2016	clinical material	PRJEB40828	Germany	2
SAMEA7451228	2017	clinical material	PRJEB40828	Germany	2
SAMN01828175	2003	I.V. Cannula	PRJNA183315	Czech Republic	2
SAMN03160612	2013	Hospital	PRJNA266271	Denmark	2
SAMN03160613	2013	Hospital	PRJNA266271	Denmark	2
SAMN03577729	2013	Decubitus pressure sore	PRJNA282640	Sweden	2
SAMN08398912	2013	respiratory sample	PRJNA431710	Spain	2
SAMN08398950	2014	respiratory sample	PRJNA431710	Italy	2
SAMN08398949	2014	respiratory sample	PRJNA431710	Greece	2
SAMN08398921	2013	respiratory sample	PRJNA431710	Spain	2
SAMN08398910	2013	respiratory sample	PRJNA431710	Spain	2
SAMN08398934	2014	respiratory sample	PRJNA431710	Spain	2

SAMN14605983	na	Human sample	PRJNA625727	Finland	2
SAMN17915434	2010	fistula	PRJNA701882	Poland	2
SAMN17915435	2010	bronchial secretion	PRJNA701882	Poland	2
SAMEA5396116	2012	catheter swab	PRJEB31555	Germany	2
SAMEA5396122	2012	screening swab	PRJEB31555	Germany	2
SAMEA5396117	2014	bronchial secretion	PRJEB31555	Germany	2
SAMEA5396115	2014	stoma swab	PRJEB31555	Germany	2
SAMEA5396118	2012	wound swab	PRJEB31555	Germany	2
SAMEA7451239	2015	clinical material	PRJEB40828	Germany	2
SAMN08159977	2017	blood culture	PRJNA427128	Italy	2
SAMN08398914	2013	respiratory sample	PRJNA431710	Italy	2
SAMN08398915	2013	respiratory sample	PRJNA431710	Italy	2
SAMN08398942	2014	respiratory sample	PRJNA431710	Greece	2
SAMN08398926	2013	respiratory sample	PRJNA431710	Greece	2
SAMN08398923	2013	respiratory sample	PRJNA431710	Italy	2
SAMN08398941	2014	respiratory sample	PRJNA431710	Greece	2
SAMN08398944	2014	respiratory sample	PRJNA431710	Greece	2
SAMN08398952	2014	respiratory sample	PRJNA431710	Greece	2
SAMN08398939	2014	respiratory sample	PRJNA431710	Greece	2
SAMN08398940	2014	respiratory sample	PRJNA431710	Greece	2
SAMN08398938	2013	respiratory sample	PRJNA431710	Greece	2
SAMEA5226469	2018	Sputum	PRJEB30134	United Kingdom	2
SAMEA5396097	2012	wound swab	PRJEB31555	Germany	2
SAMEA5396105	2013	wound swab	PRJEB31555	Germany	2
SAMEA7451221	2016	clinical material	PRJEB40828	Germany	2
SAMEA7451240	2015	clinical material	PRJEB40828	Germany	2
SAMEA5396109	2012	tracheal secretion	PRJEB31555	Germany	2
SAMN01087912	1991	urine	PRJNA183334	Czech Republic	2
SAMEA5396120	2013	drainage liquid	PRJEB31555	Germany	2
SAMEA5396124	2013	bronchial secretion	PRJEB31555	Germany	2
SAMEA5396119	2013	tracheal secretion	PRJEB31555	Germany	2
SAMEA5396121	2013	tracheal secretion	PRJEB31555	Germany	2
SAMN08398917	2013	respiratory sample	PRJNA431710	Greece	2

SAMN08398957	2014	respiratory sample	PRJNA431710	Greece	2
SAMN08398943	2014	respiratory sample	PRJNA431710	Greece	2
SAMN08398933	2014	respiratory sample	PRJNA431710	Greece	2
SAMN08398931	2013	respiratory sample	PRJNA431710	Greece	2
SAMN08398932	2013	respiratory sample	PRJNA431710	Greece	2
SAMEA6451101	2018	screen	PRJEB30134	United Kingdom	2
SAMN03160611	2013	Hospital	PRJNA266271	Denmark	2
SAMN08398913	2013	respiratory sample	PRJNA431710	Spain	2
SAMN08398909	2012	respiratory sample	PRJNA431710	Spain	2
SAMN08398958	2015	respiratory sample	PRJNA431710	Italy	2
SAMN16541575	2010	blood	PRJNA671692	Hungary	2
SAMN16541582	2017	bronchial	PRJNA671692	Hungary	2
SAMN16541580	2010	bronchial	PRJNA671692	Hungary	2
SAMN16541579	2010	bronchial	PRJNA671692	Hungary	2
SAMN16541586	2017	bronchial	PRJNA671692	Hungary	2
SAMN16541585	2017	sputum	PRJNA671692	Hungary	2
SAMN16541583	2017	sputum	PRJNA671692	Hungary	2
